# A COVID-19 Crisis in Child Physical Fitness: Creating a Barometric Tool of Public Health Engagement for the Republic of Slovenia

**DOI:** 10.3389/fpubh.2021.644235

**Published:** 2021-03-05

**Authors:** Gregor Jurak, Shawnda A. Morrison, Marjeta Kovač, Bojan Leskošek, Vedrana Sember, Janko Strel, Gregor Starc

**Affiliations:** ^1^Faculty of Sport, University of Ljubljana, Ljubljana, Slovenia; ^2^Zavod Fitlab, Logatec, Slovenia

**Keywords:** public health policy, isolation, health promotion, obesity, physical inactivity

## Abstract

The Republic of Slovenia implements the largest longitudinal database of child physical fitness in the world–SLOfit. Slovenia has some of the most physically active children globally, and it has responded rapidly to incorporate national physical activity (PA) interventions throughout the COVID-19 pandemic. Despite aggressive campaigns to maintain PA at home, the country has seen a tremendous decrease in child physical fitness over the past several months as self-isolation measures have been mandated by national authorities. These trends prompted researchers to create a method of tracking and communicating government decisions which have a direct impact on child physical activity, fitness and overall health (i.e., the SLOfit Barometer). The research team assembled experts for consultation on creation of the SLOfit Barometer after processing preliminary data on 20,000 schoolchildren which found the greatest decline in child physical fitness since systematic testing began more than 30 years ago. Only 2 months of self-isolation erased over 10 years of hard-fought health gains acquired from national public health policies and PA interventions. This crisis in child fitness requires integrated community participation and a robust public health policy response, and with the SLOfit Barometer acting as a national bellwether system, it is envisioned that policy makers and the public will advocate for bold, progressive actions to combat this national health emergency. This surveillance tool tracks government action to combat the increasing child physical inactivity and obesity trends brought on as a direct result of COVID-19 isolation regulations.

## Introduction

It has been well-established that physical inactivity contributes to increased health risk for developing a multitude of chronic diseases (e.g., cardiovascular disease, diabetes, obesity) (1). Indeed, the direct health care costs in Europe attributed to physical inactivity will reach $11.7 billion per year, with an additional $3.8 billion due to poor worker productivity (2). The actual human health toll will likely be far costlier, particularly considering these estimates do not include the unprecedented changes to human movement patterns that have occurred during the self-isolation and government-mandated physical-distancing measures enacted throughout the COVID-19 pandemic. There is now an abundance of literature investigating how self-isolation affects movement behaviors, including physical activity and sedentary time (3–6), sleep (7, 8), nutrition (6, 9), and mental health (10, 11), among others. Especially concerning are the dramatic changes to physical activity and fitness reported in the pediatric population (12, 13). Indeed, there are significant, measurable declines in the cardiorespiratory fitness of otherwise healthy children due to the deconditioning effect of the COVID-19 pandemic (14), although results on systematic fitness testing of children remain rare, likely due to the difficulty in directly testing children under pandemic circumstances, and the relative rarity of systematic, annual fitness testing occurring in children at a population level.

For over the past 30 years, the vast majority of Slovenian schoolchildren (aged 6 to 19 years) participate in a nation-wide, school-based physical fitness surveillance programme, “SLOfit” (15). In addition to providing students and their parents feedback on their child's physical and motor development and associated health-risk(s), this system allows teachers and researchers access to high-quality, standardized data on physical fitness, which is then used to directly inform public policy. Slovenia has some of the most consistently physically active children in the world (16), due in large part to the amount of physical activity (PA) time they receive within the public school system (17). When Slovenia declared an epidemic on March 12, 2020 and effectively closed all workplaces, schools and other public services (18), a national plan was activated by Slovenia's National Public Health Agency, NIJZ. Immediately following this declaration, researchers from the University of Ljubljana's SLOfit team considered how school and sport club closures would impact on children's ability to continue accessing quality, structured PA.

First, SLOfit researchers swiftly mobilized a multi-disciplinary task force consisting of experts in kinesiology, sport science, environmental physiology, pedagogy, medical doctors, and epidemiologists. This team drafted national PA guidelines during the COVID-19 pandemic (19) within 5-days of the government's call for strict self-isolation. Slovenia also implemented several actions promoting PA during self-isolation via a national campaign #vadidoma, and #trenirajdoma, described in detail elsewhere (20) but briefly, national televised home activities, radio promotions, online platforms, school systems and child health at home were all targeted and engaged daily for the duration of the most extensive “lockdown” period (roughly 2 months from March to May, 2020). Notably, the task force was apprehensive of using the slogan #stayhome which was proposed early in the process; it was thought the public would interpret this message in such a way that they should remain within the 4 walls of their home, effectively confining themselves to a sedentary capsule; thus, SLOfit overtly communicated across all mediums that people should be physically active, and outside as much as possible. The SLOfit team emphasized that countries would need to conduct direct fitness assessments on their children at a population level (20) as soon as it was safe and possible to do so.

Slovenia began experiencing a second wave of COVID-19 infection rates and further government restrictions from 19 October, 2020 until the time of writing (06 Feb, 2021). The breadth and nature of these rolling closures on PA for all citizens have been profound. Physical school closures for all children from age 6, and including kindergardens (aged 11 months to 6 years), have been in place across the country, effectively since the dates listed above. All sport facilities are closed to the public (except for professional / elite athletes), whilst playing on playgrounds and other physical structures in parks is limited. There must be greater national and international sensitivity to how restrictive measures impact human movement (20, 21). Indeed, a recent article investigating non-viral parameters associated with death rate, researchers found that the national criteria most associated with death rate were: life expectancy (and its slowdown), a public health context [e.g., metabolic and non-communicable diseases (NCD) burden vs. infectious diseases prevalence], the economy (e.g., growth national product, financial support), and the environment (e.g., temperature) (22). Enforcing strict measures to fight the COVID-19 pandemic, including lockdown, were not linked with death rate, and moreover, the authors suggested that a better understanding of the inherent factors which affect COVID-19 mortality may improve prevention strategies, one of which was highlighted as increasing population immune resilience through better physical fitness (22).

## Context

### Setting and Population

**Figure 1:**
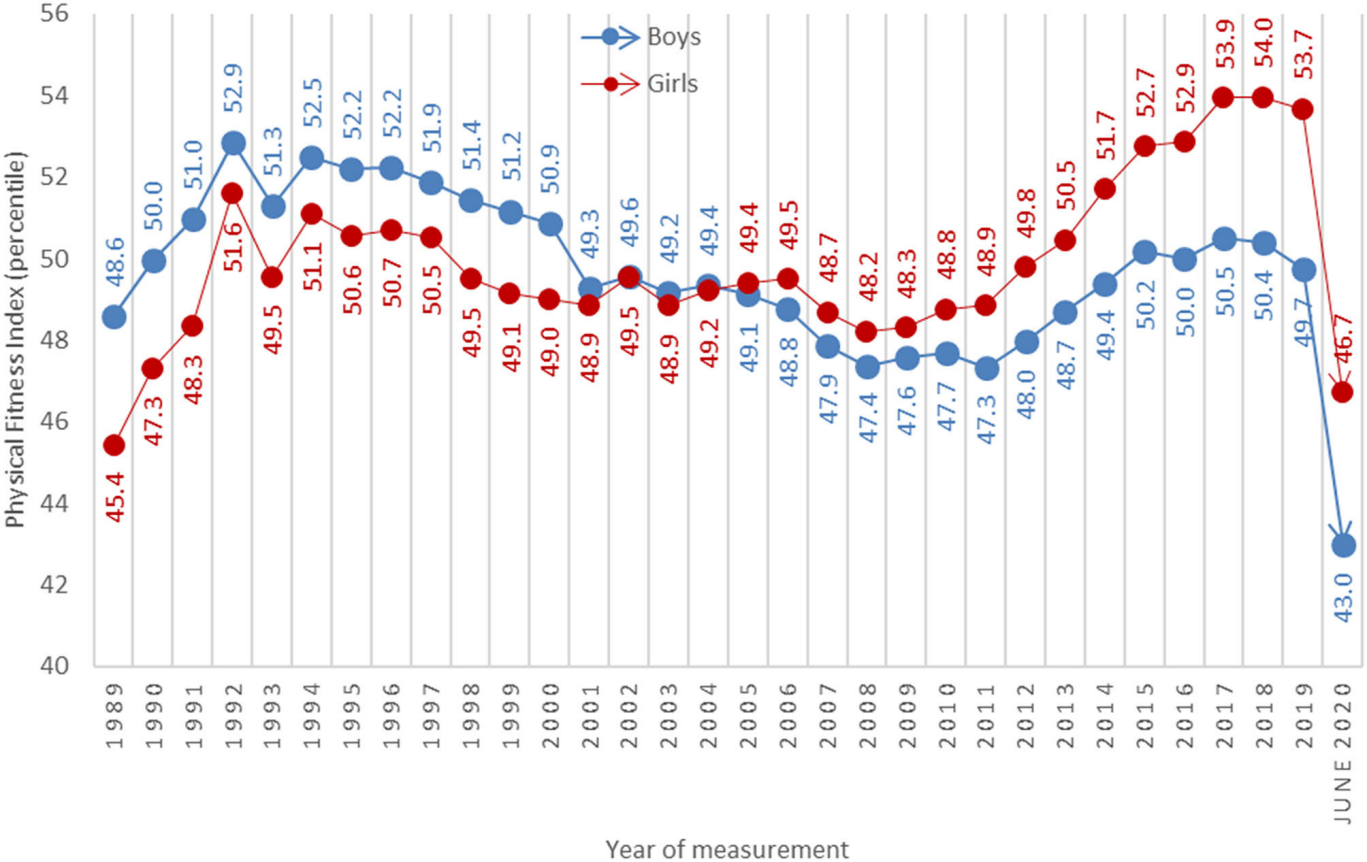
Secular changes in physical fitness index scores for Slovenian schoolchildren from 1989 to 2020. Note: All test measurements were carried out on a population level every April, whereas the 2020 measurements were carried out in June. Data for 2020 included ~15% of the entire population of 6- to 15-year-olds.

After Slovenia declared an “end” to the first wave of the epidemic on 15 May 2020, certain physical distancing measures were relaxed, allowing students to gradually return to school. Due to school closures, the regular annual SLOfit testing could not be carried out in April, but there were 20 000 students aged 6–14 years who completed fitness measurements from mid-May to June, demonstrating some exceptional school organization and commitment on the part of the individual PE teachers. The remaining schools made their measurements for the 2019/20 school year as soon as it was possible to do so, completing all testing by September 2020. Preliminary results indicate there were substantial declines in all fitness components for both boys and girls. The overall locomotor efficiency of the average Slovenian child fell by more than 13% after a two-month restriction of movement (Figure 1).

Endurance saw the greatest decline from all metrics, as did whole-body coordination. The smallest decline occurred in explosive power, although results were still worse than previous years. The full dataset are being prepared for publication, but the SLOfit team felt it was extremely important to communicate the magnitude of these negative trends as quickly as possible, and to caution other countries on underestimating the assessment of negative effects based on evidence gathered by PA questionnaires alone. It was also important for Slovenia to present a novel strategy to counteract these negative health trends so that other countries may recognize the urgency needed to advocate for quality PA of children worldwide.

## Details

### Key Programmatic Elements

**Figure 2:**
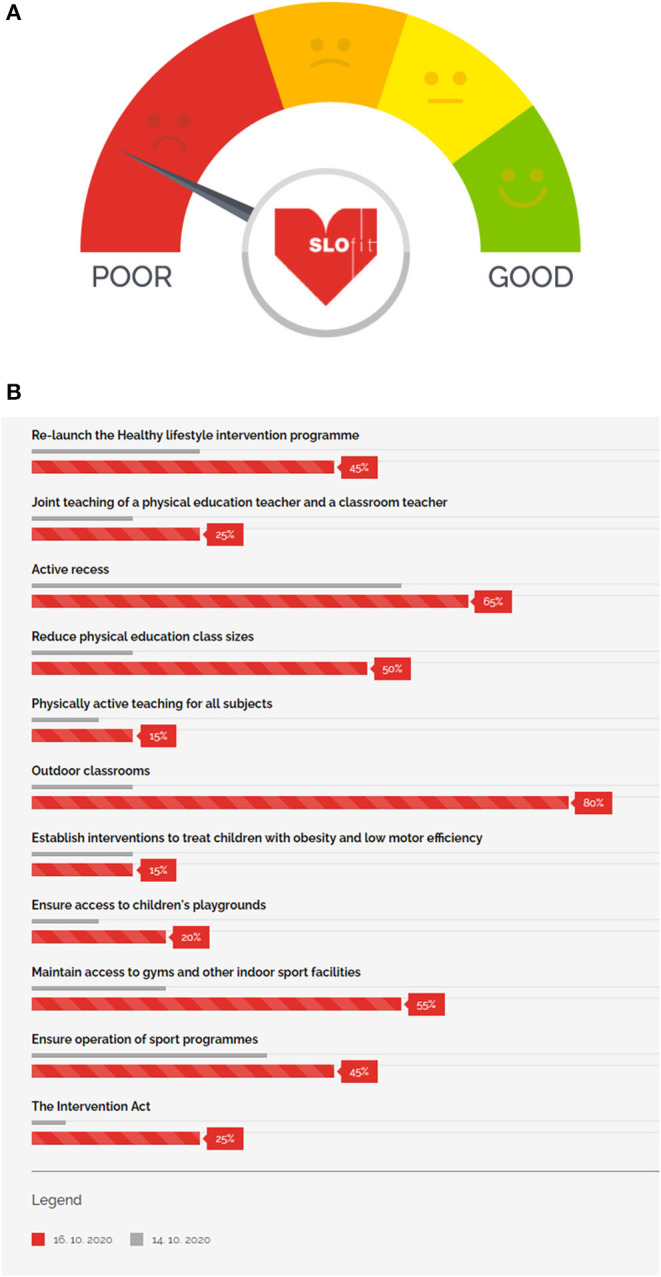
The SLOfit Barometer for public health engagement. The barometer **(A)** is an interactive dial such that the needle will move left or right to indicate progress based on 11 indicators. These indicators **(B)** are listed graphically and visible once the user clicks on the barometer logo. The user can see in greater detail which measures have been previously adopted (gray bars) and which measures are currently adopted (red bars). Data are used to monitor the successful implementation of countermeasures aimed at actively reducing sedentary behavior and poor physical fitness performance in children.

The SLOfit Barometer for Public Health Engagement is a public health tool used to monitor the successful implementation of countermeasures which are aimed at actively reducing sedentary behavior and declining physical fitness performance in children (Figure 2). The SLOfit Barometer has been developed with support from the Slovenian Olympic Committee and National Training Institute Planica (School sport partner). This involved assembling the multidisciplinary task force to give feedback on whether presenting a communication tool directly to the public would be beneficial for promoting and advocating for PA to be considered at the national government policy-making level. After members responded in the affirmative, specifications were sent to a designer who developed a user-friendly, simple, intuitive web design. Finally, team members liaised with designers to create an interactive-supported feature which enables the dial to move depending on policies adopted, and based on 11 key indicators (specified in detail below). The SLOfit Barometer has been prominently placed on the SLOfit homepage of the Faculty of Sport, University of Ljubljana website, immediately visible to every visitor. After clicking on the logo, a subpage opens, presenting countermeasure details and how close a given item is to full deployment. Health care professionals and the public also participate in the Barometer assessment through surveys, the results of which are published on the SLOfit website and promoted through social media channels to attract further participation. These documents form the basis of the barometric assessments. Each countermeasure is classified into one of the following 11 items, communicated graphically:

1. Re-launch the Healthy lifestyle national intervention programme. SLOfit researchers are demanding an immediate re-launch of previously well-proven “Healthy Lifestyle” intervention programme which substantially increased child motor efficiency, decreased prevalence of obesity and lowered class absenteeism due to illness (23).
2. Joint teaching of PE by a PE specialist and the classroom teacher. More frequent and better-quality PA should be provided to young students from grades 1 to 5. Introducing additional joint-teaching PE classes would increase the frequency of PA and its intensity with PE teacher competencies, including lower students numbers per teacher (23).
3. Active recess. All schools should be mandated to provide at least a 20-min of active recess each day, preferably outdoors, to all students. During recess, schools should respect the current restrictions on maintaining physical distance, e.g., school “bubble” groups.
4. Reduce PE class sizes. Large differences in motor performance require appropriate differentiation in PE classes, for groups involving children with special needs, health and developmental problems, as well as individual approaches. Thus, changes from current norm of 20 children per PE class should be reduced to 16, to enable better quality teaching, greater safety and less possibility of virus transmission.
5. Physically active teaching for all subjects. Teachers should be encouraged, and educated to, abandon the prevailing, sedentary style of learning and adopt appropriate movement in for all learning contents.
6. Outdoor classrooms. Schools should incorporate outdoor learning strategies into the classroom in order to increase exposure to green spaces, physical activity, vitamin D and the positive benefits such exposures exude. Instead of focusing on training teachers for on-line classes, emphasize should be on how to deliver course content efficiently outdoors.
7. Establish interventions to treat children with obesity and low motor efficiency. Based on previous experience from pilot projects, and in cooperation with the school and health sector, diagnosing children with obesity and poor physical fitness for national intervention programmes must be a national priority.
8. Ensure access to children's playgrounds. Playgrounds should remain open even in the event of an epidemic declaration, with special conditions of use (e.g., play within household members, disinfect hands before using equipment, etc.).
9. Maintain access to gyms and other indoor sport facilities. Management of sport premises should ensure open access to school and out-of-school users and describe under which special conditions people can be allowed to use them.
10. Ensure operation of sport programmes. There should be no lockdown of sport programmes. The government, in cooperation with Slovenian Olympic Committee, should enable sport programmes to continue by determining conditions for safe practice.
11. The Intervention Act. Secure legal framework and finances to ensure the proposed countermeasures are adopted at the state and local level in Slovenia.

## Discussion

### Practical Implications

With the cooperation of SLOfit and participation from Slovenian Olympic Committee and National Training Institute Planica, we have created a national surveillance tool for the improvement of physical fitness in Slovenian schoolchildren. The SLOfit Barometer of Public Health Engagement is being used as a pressure gauging tool for tracking government commitments to invest more heavily in their public health system, specifically for children. It fosters an environment of public accountability, so all citizens may understand the value of maintaining adequate human movement for overall health, especially as the COVID-19 pandemic continues to evolve, and isolation measures continue to impact the daily life of the global citizenry. The practical implications of creating such a tool are to enable researchers, public health authorities, education experts and the general public to have a standardized tool with which to observe, track, and demand objective changes in public health policy, as it relates to positively affecting child health during this global pandemic.

### Surveying the Public on Barometric Measures

Since the first week of “lockdown” restrictions began, follow-up questionnaires have been distributed across social media platforms and via established links with schools across Slovenia using the SLOfit Barometer to track progress on various Barometer benchmarks. One example is described in detail below.

SLOfit researchers were interested in quantifying how well-professionals between schools and doctors conduct best-practice communication between themselves, the team prepared an online survey within the SLOfit Barometer monitoring tool, based on recommendations for participation. The SLOfit online app has been used by 216 primary schools in 2020. Based on their experience from previous projects, researchers asked teachers a range of questions, including whether they regularly cooperated with doctors in dealing with physically less efficient and obese children, whether they used the “My SLOfit application” themselves, and if so, in what way. It was found that the number of cooperation practices between schools, PE teachers and doctors was decidedly too small, with only 20% claiming that most children receive this type of holistic, coordinated treatment between professions. Interestingly, when explaining why schools do not work with doctors more closely, teachers most often answered that schools do not have a dedicated school doctor. In only one private school (which does not have a permanent school district), this type of answer was possible, otherwise schools are supposed to have a defined school doctor. The questionnaire highlighted that in many environments, even basic communication between education and health care is not so well-established, and that teachers do not always know to what extent they have access to medical expertise for their pupils.

These data have provided researchers with important information on how to encourage more schools and health centers to better communicate with each other at the local level. Because there was still a great deal of work to do in this area, and because data on providing coordinated approaches to child health are still lacking in public schools, the benchmark (#7) was not improved on the SLOfit Barometer (i.e., “Establish interventions to treat children with obesity and low motor efficiency”) according to this survey alone. But, by presenting the results of this survey to national news media, researchers can advocate that all children deserve a collaborative approach to systematic treatment within the public-school and health system. Easy, reliable access to healthcare via public schools will become particularly important in the light of the current decline in physical efficiency and the rise in childhood obesity in 2020-the consequences of distance schooling, interruptions in sports activities and restrictions on physical movement. The lessons learned from creating the SLOfit Barometer have been that only with an active approach will we be able to neutralize the negative consequences of inactivity, and protect the younger generations from increased morbidity, poorer learning abilities, low productivity, and poor well-being.

### Practical Public Policy Outcomes

Out of these initiatives, there have been 3 significant outcomes. First, the SLOfit proposal for countermeasures to inactivity during self-isolation were endorsed by an Expert Council of Government for Sport and participants of the national congress “Sport for All.” The measures were adopted on 28.09.2020 when the council endorsed the proposals. This affected all indicators of the SLOfit Barometer, increasing each benchmark by 2%. Secondly, at the “Sport for All” congress, held from 27.11.2020 to 28.11.2020, the congress concluded with a policy brief to the Government of the Republic of Slovenia to include sport experts on the COVID-19 expert group when adopting future measures to prevent the spread of the virus, and to take into account PA aspects (e.g., importance of PA on physical, psychological and social health), including formulation of any future public health measures. At the same time, the Congress proposed to the Government that promoting PA in their own public appearances was important, and in accordance with the current restrictions against the spread of the virus. Whilst improving the epidemiological picture in the country and releasing measures in the country, the Government was also encouraged to promote PA to the population at large by offering subsidies/facilitations for inclusion in sports exercise programs. The answer to this brief is forthcoming. Finally, the SLOfit team has suggested that the Slovenian Olympic Committee launch a national campaign #ExerciseResponsibly with these key messages:

- the immune system can be strengthened by regular physical exercise, including during lockdown, but adjusted according to limitations;
- if possible, practice and exercise outside, or in nature
- the key challenge of responsible exercise during the spread of infection is to maintain adequate distance between the trainee and others;
- practice consistent and persistent cough and hand hygiene during sports training (even when there is no outbreak of infections);
- promoting personal responsibility for self-isolation in the case of suspicion of contact with an infected person (certainly, do not practice in a group setting).

Practical recommendations on maintaining an active lifestyle during confinement periods (e.g., home-based exercise, physical activity outdoors, etc) are available from several peer-reviewed sources (24), including a systematic review (25), from Slovenia (19) and recommendations specifically for children (13).

## Study Considerations

The SLOfit Barometer is visible on the University of Ljubljana's SLOfit team website and regularly promoted via posts on social media platforms (e.g., Facebook). Although these online outlets see a great deal of traffic from the public, it may not reach certain demographics across all of Slovenia. The researchers understand that there may be a positive bias to any questionnaires distributed via online and social media sources since these online tools may not be as easily accessible to participation from those of lower socio-economic status, or those who have limited internet access. The SLOfit Barometer intervention is the beginning of a concerted and systematic effort to bring sustained pressure on those who manage and create public policy surrounding national public health initiatives brought on by the COVID-19 global pandemic. As such, it is a work-in-progress, and input from all stakeholders (Olympic committee, educational partners, public-at-large) will be critical in guaranteeing its effectiveness to bring about meaningful health policy changes for protecting child health and ensuring equal access to continued quality, professionally-led physical activity. The SLOfit Barometer has not been implemented in other countries and has been developed within the social construct and pedagogical infrastructure of the Republic of Slovenia in mind. It is the authors' hope that the perspectives brought forth in this communication will spur discussion both within and outside of the country. The intention of this work is to prompt other countries to consider developing similar communication tools for their government based on the specific needs and challenges of their own region, country, and society.

## Data Availability Statement

The raw data supporting the conclusions of this article will be made available by the authors, without undue reservation.

## Ethics Statement

All the aspects of data gathering and analysis of the SLOfit data were reviewed and approved by the National Medical Ethics Committee of the Republic of Slovenia (ID 102/03/15). Written informed consent to participate in this study was provided by the participants' legal guardian/next of kin.

## Author Contributions

GJ provided concept and design. GJ and SM drafted the work, GS gathered and analysed the data, GS, MK, BL, VS, and JS provided substantial feedback on content and edits. All authors contributed to the writing of paper and approved the final version before manuscript submission.

## Conflict of Interest

JS is founder of Zavod Fitlab, a fitness-based private institute based in Logatec, Slovenia and business unit Arcus Medici which prepares exercises on an outpatient clinical setting basis. The remaining authors declare that the research was conducted in the absence of any commercial or financial relationships that could be construed as a potential conflict of interest.
